# Clockwise aortic root rotation in patients with tetralogy of fallot assessed with cardiovascular magnetic resonance imaging

**DOI:** 10.1007/s10554-025-03438-2

**Published:** 2025-06-23

**Authors:** Marco Voortman, Jan van Es, Clemens von Birgelen, Eline H. Ploumen, Carine J. M. Doggen, Lodewijk J. Wagenaar

**Affiliations:** 1 Department of Cardiology, Thoraxcentrum Twente, Medisch Spectrum Twente, Koningsplein 1, Enschede, 7512 KZ The Netherlands; 2 Department of Health Technology and Services Research, Faculty of Behavioural Management and Social Sciences, Technical Medical Centre, University of Twente, Enschede, The Netherlands

**Keywords:** Tetralogy of fallot, Magnetic resonance imaging, Aortic root

## Abstract

Rotation of the aortic root (AoR) has previously been observed in children with Tetralogy of Fallot (TOF) using 2-dimensional echocardiography. The present study uses cardiovascular magnetic resonance (CMR) imaging to assess whether a clockwise AoR rotation is a common feature in adults with TOF. AoR rotation was measured in the CMR images of consecutive adult patients with corrected TOF and controls. The AoR rotation was assessed in the parasternal short-axis view by measuring the angle of the non-coronary sinus (angle NCS) relative to the interatrial septum (IAS). The angle between IAS and posterior atrial wall was measured to asses IAS reliability as a key anatomical landmark. Inter-observer agreement was determined using intra-class correlation coefficients (ICC). A total of 25 adults with TOF (mean age:33.2 ± 13.1 years;52% male) and 30 controls (mean age:54.5 ± 19.0 years;50% male) were assessed. The angle NCS was greater in TOF patients than in controls (46.2 ± 17.1⁰vs.23.4 ± 15.0⁰, *p* < 0.001), reflecting a clockwise AoR rotation. As expected, between TOF patients and controls there was no significant difference in the angle IAS (81.1 ± 8.4⁰vs.78.4 ± 9.5⁰, *p* = 0.46). Inter-observer reliability of CMR measurements was good for the clinically important angle NCS (ICC 0.77, 95%-CI:0.63–0.86) and moderate for the angle IAS (ICC 0.68, 95%-CI:0.51–0.80). This is the first study to demonstrate with CMR that a clockwise rotation of the AoR is a common feature in patients with TOF. This clinical investigation shows the value of CMR for the assessment of the spatial AoR position in adult patients with congenital heart disease.

## Introduction

Tetralogy of Fallot (TOF) is the most prevalent cyanotic congenital heart disease, with many TOF patients having a favourable long-term survival after surgical correction [1, 2]. TOF is characterized by a ventricular septal defect, obstruction of the right ventricular outflow tract, right ventricular hypertrophy, and overriding aortic root [3]. Moreover, aortic root dilation is a common feature in TOF [4, 5]. In addition to these key morphological features, a clockwise rotation of the aortic root (AoR) was seen in a previous study with 2-D echocardiography that compared children with TOF and healthy controls [6]. More recent investigations with advanced imaging techniques, such as computed tomography, have reported variations in AoR rotation in the general population and associations between a clockwise rotation and both aortic dilation and dissection [7–9].

Cardiovascular magnetic resonance (CMR) imaging provides 3-D image data sets with a high spatial resolution and permits non-invasive assessments without iodine contrast of both, cardiac structures and the aorta [10]. In patients with TOF, CMR is used for surveillance including the detection of potential late cardiovascular complications [11]. In addition, CMR is suitable for the assessment of AoR, as recently demonstrated by a validation study in healthy subjects [12].

To date, CMR has not yet been used for assessing AoR rotation in adult patients with congenital heart disease. Therefore, in the present study we examined both adults with corrected TOF and healthy controls to assess whether a clockwise rotation is as a common feature in patients with TOF.

## Methods

### Study population and data collection

We reviewed the electronic medical records to identify a series of consecutive adult patients with corrected TOF who underwent CMR imaging of the short-axis plane at the aortic valve level at our hospital from January 1, 2011 until December 31, 2023. In addition, we assessed a control group of 30 consecutive adult patients who (1) underwent CMR for indications other than suspected or known congenital or aortic diseases (from November until December 2020) and (2) had no history of cardiovascular surgery or known connective tissue disease. None of the TOF patients had aortic coarctation. The only exclusion criterion was poor visualization of the anatomical landmarks, for example, as a result of arrhythmias or motion artefacts. Anonymized data were collected of all TOF patients and controls, including several patient demographics such as age, gender, body surface area and type of TOF repair. In addition, data of the aortic root diameter were collected. These measurements were taken at the level of sinus of Valsalva, based on largest cusp-to-cusp measurement of the sinus during end-diastole. This single-centre study adhered to the Declaration of Helsinki (2013 revision) and received approval from the Medical Ethics Review Committee of Medisch Spectrum Twente, Enschede, and the requirement for obtaining individual informed consent was waived.

### CMR technique and imaging analysis

CMR images were acquired using a 1.5 Tesla magnetic resonance scanner (Ingenia Ambition, Philips Medical Systems Best, the Netherlands). The standard protocol included velocity-encoded cine gradient echo imaging with a slice thickness of 8 mm, spatial resolution of 1.2 × 1.2 mm, temporal resolution of 26 milliseconds, velocity encoding at 200 cm/sec, and 30 cardiac phases throughout the cardiac cycle. Images were obtained during a breath-hold at expiration and analysed using dedicated post-processing software (Philips Intellispace Portal 10.0; Philips Healthcare) for precise geometric measurements and calculations.

### Measurements of AoR rotation angles

**Fig. 1:**
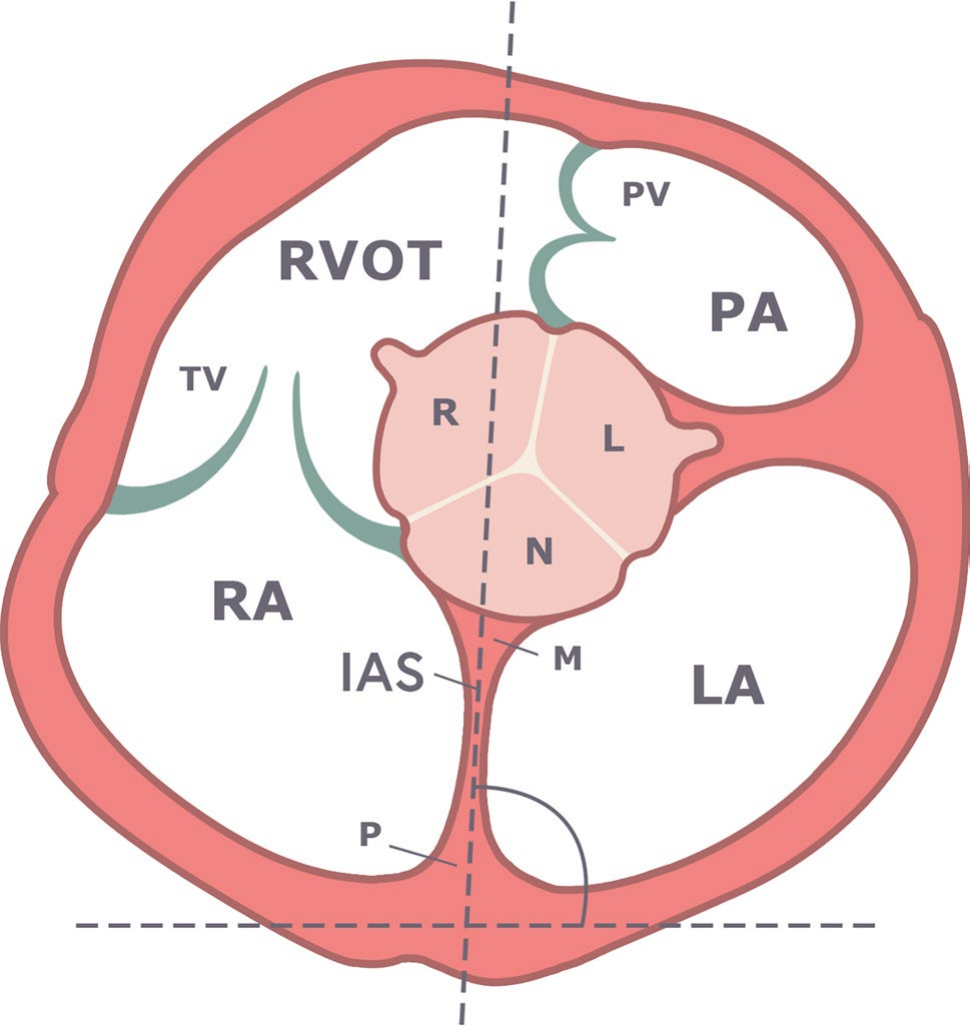
Schematic representations of the short-axis of the aortic valve with the construction of the angle of the interatrial septum (IAS). The axis of the IAS was considered the reference line and determined by its anterior and posterior insertions, representing the muscular buttress (M) and the posterior interatrial infolding (P). Abbreviations: RVOT, right ventricular outflow tract; PV, pulmonary valve; R, right coronary sinus; L, left coronary sinus; N, non-coronary sinus; RA, right atrium; LA, left atrium; IAS, interatrial septum; Z, the right coronary-left coronary commissure of the aortic valve; PA, pulmonary artery; TV, tricuspid valve; AoR, aortic root; M, anteroinferior muscular buttress; P, posterior interatrial infolding

The CMR-based AoR measurements were performed according to an approach that has previously been validated and described in detail [12]. AoR rotation was measured using the angle of the non-coronary sinus (angle NCS), defined by the axis of the interatrial septum (IAS) and a line mid-point of the NCS and the commissure between the right and left coronary sinus. Figure 1 displays a schematic representation of the short-axis plane at the aortic valve level with construction of lines to define the axis of the IAS. This anatomical landmark was previously shown to be feasible as a cardiac reference point in healthy subjects [12]. In order to assess whether this also applies to TOF patients, we measured the IAS angle in TOF patients and controls, and compared the findings. Inter-observer reliability for AoR root rotation measurement was evaluated by two examiners (M.V., cardiologist in training, and J.E., MRI imaging physician). Both of the examiners were provided with a schematic diagram and a written description of the CMR approach, and they were blinded to each other’s measurements.

### Statistical analysis

Continues variables were reported as mean ± standard deviation, if normally distributed, or as median (interquartile range). Between group differences of normally distributed variables were assessed were using the Student’s t-test. Categorical variables were reported as number and percentage, and between group difference were assessed with the chi-square test. The Intraclass correlation coefficients (ICC) with 95% confidence intervals were calculated to assess inter-rater reliability. The ICC values were interpreted as follows: below 0.5 (poor reliability), 0.5–0.75 (moderate reliability), 0.75–0.9 (good reliability), and greater than 0.90 (excellent reliability) [13]. Bland–Altman plots were used to visualize the agreement between the rotation measurement obtained by two analyst (interrater reliability) to rule out systematic difference between the results [26]. Heteroscedasticity was investigated through visual inspection. Two-sided p-values and confidence intervals were considered, with statistical significance set at *p* < 0.05. The statistical analyses were conducted using SPSS version 24 (IBM, Armonk, New York).

## Results

A total of 26 consecutive TOF patients and 30 controls were identified and assessed. One TOF patient was excluded due to poor CMR visualization of the anatomical structures due to motion artefacts.

Ultimately, the study cohort comprised 25 patients with TOF, who at the time of CMR imaging had an age of 33.2 ± 13.1 years, and 12 (48%) were men. The median patient age at the time of primary repair was 2 years (range 0–31 years). The vast majority of all patients (24, 96%) had TOF with pulmonary stenosis; of these patients 12 (48%) underwent transannular RVOT patch surgery and 13 (52%) valve-sparing repair. One patient had a right ventricle–to–pulmonary artery conduit repair, while the exact pulmonary artery morphology was unknown. In TOF patients, the AoR diameter was ≥ 40 mm in 11 (44%) patients. Of the 30 controls (age: 54.5 ± 19.0 years) 15 (50%) were men. In controls, the AoR diameter was ≥ 40 mm in 3 (10%) patients. Baseline demographics and patient characteristics of both the study and the controls are presented in Table 1.

**Table 1:** Patient characteristics

Parameter	TOF Patients (*n* = 25)	Controls (*n* = 30)	*P* value
Age, years (range)	33.2 ± 13.3 (12–64)	54.5 ± 19.0 (16–79)	< 0.001
Male sex, n (%)	12 (48)	15 (50)	0.88
Body surface area, m^2^	1.8 ± 0.2	2.0 ± 0.2	< 0.001
Aortic Root diameter, mm	38.6 ± 6.4	33.7 ± 4.5	0.002

*TOF* Tetralogy of Fallot

Data are n (%) or mean ± SD

**Fig. 2:**
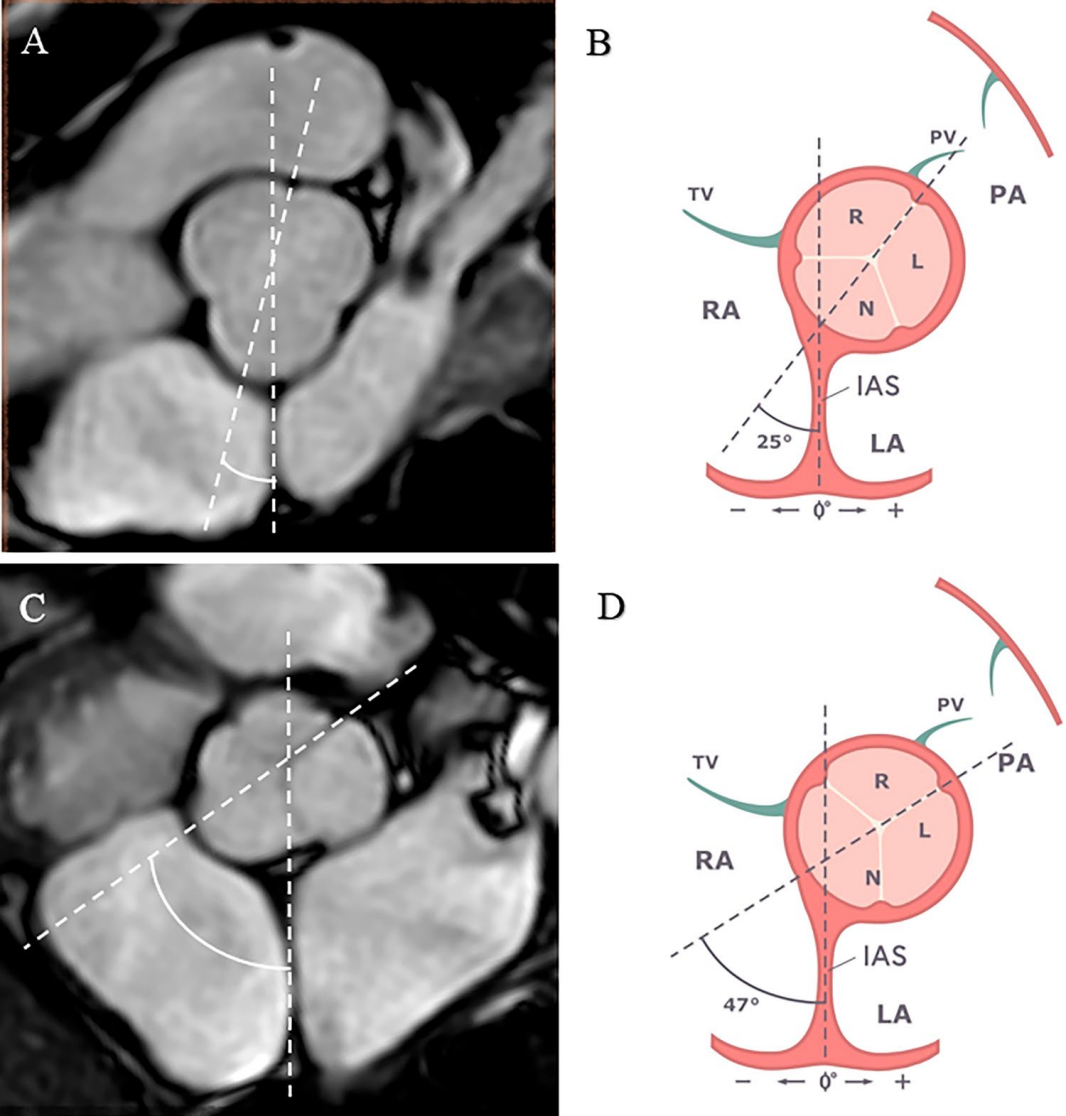
Schematic representation of the short-axis plane at aortic valve level with the construction of the angle of the NCS for measuring AoR rotation. (**A**) The short axis aortic root cine stack with a 3D BTFE sequence in controls. (**B**) Scheme defining the average angle of the NCS in controls. (**C**) The short axis aortic root cine stack with a 3D BTFE sequence in TOF patients. (**D**) Scheme defining the average angle of the NCS in TOF patients. Abbreviations: PV, pulmonary valve; TV, tricuspid valve; PA, pulmonary artery; R, right coronary sinus; L, left coronary sinus; N, non-coronary sinus; RA, right atrium; IAS, interatrial septum; LA, left atrium; AoR, aortic root; NCS, non-coronary sinus; RCS, right coronary sinus; LCS, left coronary sinus

CMR measurements are presented in Table 2. The angle IAS did not differ significantly between the TOF patients and the controls (81.1 ± 8.4⁰ vs. 78.4 ± 9.5⁰, *p* = 0.461), which permitted us to use the IAS as a reference point. The angle NCS was significantly wider in TOF patients than in the controls (46.2 ± 17.1⁰ vs. 23.4 ± 15.0⁰, *p* < 0.001). Figure 2 illustrates the difference in AoR rotation between the patients with TOF and the controls.

**Table 2:** Average measurements of rotation angles

Angle	TOF Patients (*n* = 25)	Controls (*n* = 30)	*P* value
IAS, ^o^	81.1 ± 8.4	78.4 ± 9.5	0.461
NCS, ^o^	46.2 ± 17.1	23.4 ± 15.0	< 0.001

*TOF* Tetralogy of Fallot; *IAS* Interatrial septum; *NCS* Non-coronary sinus

Data are mean ± SD

**Fig. 3:**
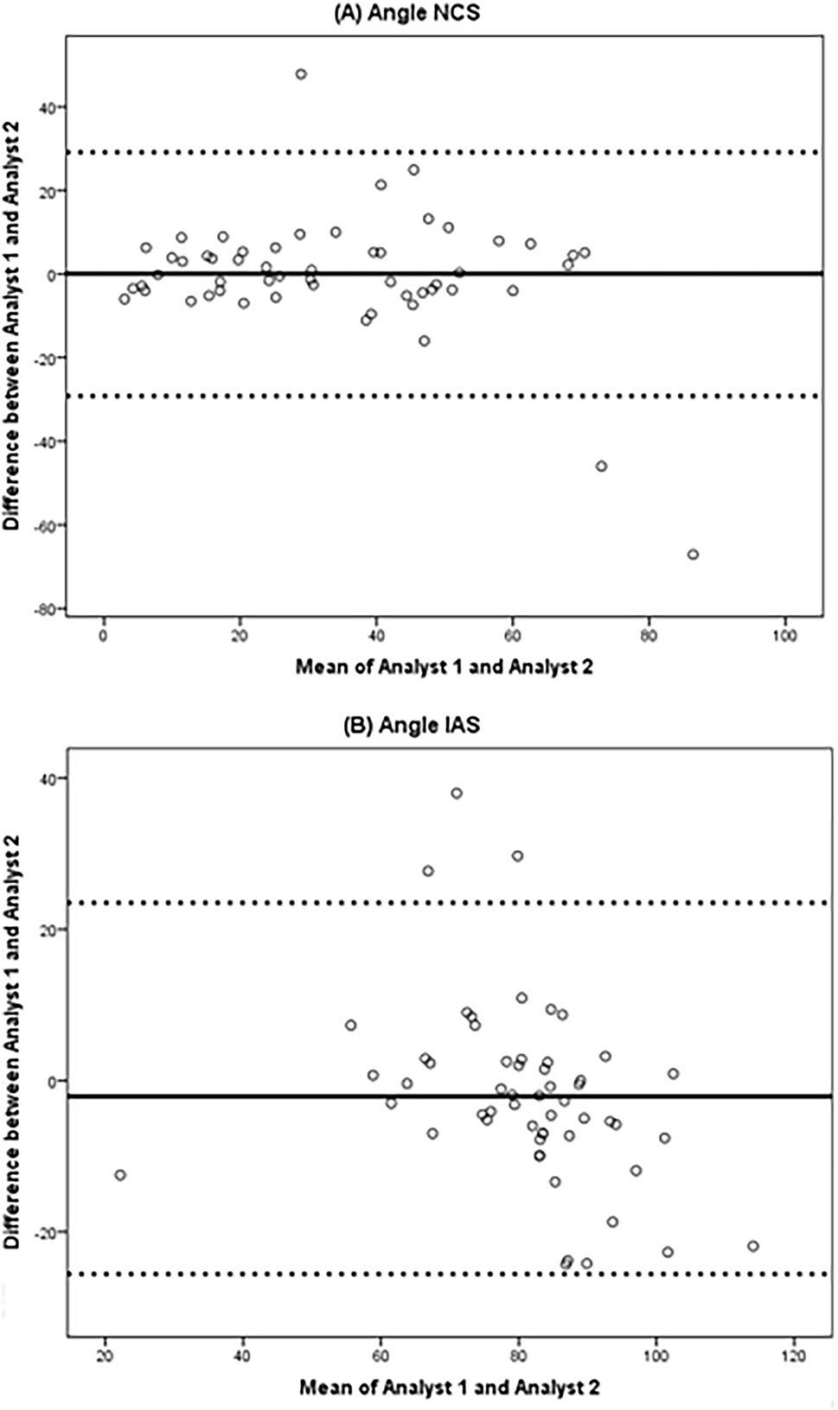
The Bland–Altman plot of interrater reliability, showing the difference and mean of (A) angle NCS, and (B) angle IAS. The difference in scores between the two assessments was plotted on the y-axis and the mean of the scores of the two assessments was plotted on the x-axis. The solid line represents the group mean difference, and the dotted lines show the 95% upper and lower limits of agreement

The results of the CMR measurements and the inter-observer reliability are shown Table 3. The interobserver reliability, the intraclass correlation coefficient of angle NCS was good (ICC 0.77, 95%-CI: 0.63–0.86). The intraclass correlation coefficient of the angle IAS was moderate (ICC 0.68, 95%-CI: 0.51–0.80). The Bland–Altman plots shows excellent average agreement and narrow limits of agreement (Fig. 3). Visual inspection of the plot did not show any heteroscedasticity, indicating that there was no systematic difference between the analysts.

**Table 3:** Average measurements and interobserver reliability of rotation angles from 30 TOF patients and 25 controls by 2 independent analysts

Angle	ICC	95% Confidence Interval	
		Lower Bound	Upper Bound
**IAS**, ^**o**^	0.68	0.51	0.80
**NCS**, ^**o**^	0.77	0.63	0.86

*ICC* Intra-class correlation coefficient; *TOF* Tetralogy of Fallot; *IAS* Interatrial septum; *NCS* Non-coronary sinus

Data are mean ± SD

## Discussion

### Main findings of the study

The present study revealed a significant difference in NCS angle between the patients with TOF and the controls, confirming the presence of a clockwise rotation of the AoR in adult patients with corrected TOF.

### Previous studies

To our knowledge, only a single cardiac imaging study, published in 1986, has systematically and quantitatively assessed the spatial position of the AoR in patients with TOF and compared it with findings in normal controls [6]. The findings of our present CMR study corroborate this previous echocardiography study that demonstrated a relevant clockwise AoR rotation in 22 children with TOF as compared to 23 controls (59.2 ± 10.7⁰ vs. 23.4 ± 8.3⁰, *p* < 0.001) [6]. Both the angle of the intra-atrial septum (IAS) and the AoR rotation angle in the control group appear to be similar in both studies. However, in TOF patients the clockwise AoR rotation measured with CMR was less pronounced than in the previous echocardiographic study. This discrepancy between the findings of the two studies might be attributed to the substantial difference in age (children vs. adults) or potential variations in TOF, showing more or less pronounced abnormalities. The latter is supported by a recent study with multislice computed tomography angiography, which found that TOF patients with atresia of the pulmonary artery exhibited a significantly more pronounced AoR rotation than those who only had a pulmonary stenosis (64.9⁰ vs. 52.6⁰) [14]. That study with computed tomography did not compare the findings in TOF patients with healthy controls, and it did not report the reliability of its reference point (i.e., the angle IAS). Nevertheless, its principal methodology appears to be similar to the approach applied in the current CMR study, and the observed AoR rotation angles in TOF patients with pulmonary stenosis aligns with our findings.

Previous computed tomography-based analyses reported smaller AoR rotation angles in healthy controls. In one study, the AoR rotation angle measured on average 15.7 ± 10.7° [15], and in another study the median AoR rotation angle was reported to be 15.5° (range: −32° to 44.7°) [8]. Most likely, the difference between these study results and the AoR rotation measurements in controls of the present study can be attributed to methodological dissimilarities such as different measurement techniques. These previous CT studies used multiplanar reformatting of the short axis of the AoR. Another factor could be the use of a different anatomical landmark as reference: the long axis of the triangular structure composed of the right fibrous trigone and the muscular buttress within the intra-atrial septum [16].

### Future perspectives and clinical implications

In patients with corrected TOF, long-term monitoring of cardiovascular anatomy is essential to detect potential aortic complications, such as aortic dilatation, formation of an aortic aneurysm, and leakage of the aortic valve [4, 5]. In the present study, a significant number of TOF patients also had an aortic diameter greater than 40 mm, which corresponds with the previously reported prevalence of aortic dilatation and aneurysm [17, 18]. The mechanisms underlying aortic dilatation following TOF repair remain poorly understood. Contributing factors may include (1) increased aortic stiffness, (2) elevated wall shear stress, and (3) abnormal flow formation throughout the ascending aorta [19–22].

AoR rotation could be another significant factor in the development of aortic complications in patients with TOF. In the general population, clockwise AoR rotation has been associated with dilation and dissection of the ascending aorta [7, 8, 15]. Similar associations have been reported in patients who have undergone an arterial switch operation for transposition of the arteries, were neo-aortic root rotational angle was found to be associated with aortic dilatation and neo-aortic valve incompetence [23]. In patients with repaired TOF, prospective studies using dedicated imaging protocols may help clarify whether, and to what extent, AoR rotation is associated to progressive aortic dilatation. Therefore, assessing AoR rotation may provide valuable insights into the underlying causes of aortic complications in TOF patients.

Importantly, the heterogeneity of the TOF phenotype, both in terms of native anatomy and surgical repair, may influence aortic root rotation [14]. However, due to missing early clinical and surgical data, subgroup analyses were not feasible. Future studies with larger and more detailed datasets may help identify which TOF subtypes are more or less prone to aortic root.

A comprehensive understanding of AoR rotation may also assist physicians in executing safe and precise procedures involving the aortic root, particularly in avoiding future problems with atrioventricular conduction [24, 25]. This knowledge can enhance surgical planning and decision-making, potentially reducing the risk of complications and improving long-term outcomes for patients with corrected TOF.

### Limitations

This study has limitations. The sample size is relatively small but similar to that of many previous imaging studies in congenital heart disease patients. Although the reliability of CMR measurements is acceptable in this study, it is lower than the previously reported excellent reproducibility of measurements with this approach in healthy subjects [12]. While the short-axis AoR cine stack provides a good imaging view in healthy subjects (assessed as the control group), it may not always optimally visualize all structures in TOF patients without multiplanar reformatting. In addition, a factor that could influence the inter-observer reliability is the extended period during which this consecutive series of TOF patients were studied with CMR. Advancements in CMR technology and analysis software, achieved during this period, may have contributed to an improvement in imaging quality and subsequently higher reported levels of ICC.

A further limitation is that aortic root rotation was described using a single angle rather than a 3D vector. While an angular measurement is straightforward and facilitates comparison across subjects, it may oversimplify the true spatial orientation of the aortic root. Future studies combining single-angle and vector-based 3D analysis may provide a more accurate and comprehensive representation, particularly in anatomically variable TOF patients.

## Conclusion

This is the first study to demonstrate with CMR that a clockwise rotation of the AoR is a common feature in patients with TOF. This clinical investigation shows the value of CMR for the assessment of the spatial AoR position in adult patients with congenital heart disease.

## Data Availability

No datasets were generated or analysed during the current study.

## Abbreviations

TOF: Tetralogy of Fallot
CMR: Cardiovascular Magnetic Resonance
AoR: Aortic Root
IAS: Interatrial Septum
ICC: Intra-class correlation coefficient
NCS: Non-Coronary Sinus

## References

[1] Ishigami S, Ye XT, Buratto E et al (2024) Long-term outcomes of tetralogy of fallot repair: A 30-year experience with 960 patients. J Thorac Cardiovasc Surg 167(1):289–302e11. 10.1016/j.jtcvs.2023.04.015 37169063 10.1016/j.jtcvs.2023.04.015

[2] Liu Y, Chen S, Zühlke L et al (2019) Global birth prevalence of congenital heart defects 1970–2017: updated systematic review and meta-analysis of 260 studies. Int J Epidemiol 48(2):455–463. 10.1093/ije/dyz009 30783674 10.1093/ije/dyz009 PMC6469300

[3] Anderson RH, Weinberg PM (2005) The clinical anatomy of tetralogy of fallot. Cardiol Young 15(Suppl 1):38–47. 10.1017/s1047951105001010 15934690 10.1017/s1047951105001010

[4] Sengupta A, Lee JM, Gauvreau K et al (2023) Natural history of aortic root dilatation and pathologic aortic regurgitation in tetralogy of fallot and its morphological variants. J Thorac Cardiovasc Surg 166(6):1718–1728e4. 10.1016/j.jtcvs.2023.04.014 37164053 10.1016/j.jtcvs.2023.04.014

[5] Siripornpitak S, Sriprachyakul A, Wongmetta S, Samankatiwat P, Mokarapong P, Wanitkun S (2021) Follow-up aortic dilatation in patients with repaired tetralogy of fallot using cardiovascular magnetic resonance. Eur J Radiol Open 8:100354. 10.1016/j.ejro.2021.100354. Published 2021 May 12 34026947 10.1016/j.ejro.2021.100354 PMC8134066

[6] Isaaz K, Cloez JL, Marçon F, Worms AM, Pernot C (1986) Is the aorta truly dextroposed in tetralogy of fallot?? A two-dimensional echocardiographic answer. Circulation 73(5):892–899. 10.1161/01.cir.73.5.892 3698234 10.1161/01.cir.73.5.892

[7] Saremi F, Cen S, Tayari N et al (2017) A correlative study of aortic valve rotation angle and thoracic aortic sizes using ECG gated CT angiography. Eur J Radiol 89:60–66. 10.1016/j.ejrad.2017.01.009 28267550 10.1016/j.ejrad.2017.01.009

[8] Tretter JT, Mori S, Saremi F et al (2018) Variations in rotation of the aortic root and membranous septum with implications for transcatheter valve implantation. Heart 104(12):999–1005. 10.1136/heartjnl-2017-312390 29146623 10.1136/heartjnl-2017-312390

[9] Miazza J, Winkel D, Thieringer F et al (2024) Aortic root rotation: morphological analysis of the aortic root with three-dimensional computed tomography. Eur J Cardiothorac Surg 65(3):ezae040. 10.1093/ejcts/ezae040 38310332 10.1093/ejcts/ezae040 PMC10931524

[10] Leonardi B, Secinaro A, Calvieri C et al (2019) The role of 3D imaging in the follow-up of patients with repaired tetralogy of fallot. Eur Rev Med Pharmacol Sci 23(4):1698–1709. 10.26355/eurrev_201902_17132 30840295 10.26355/eurrev_201902_17132

[11] Moscatelli S, Pergola V, Motta R et al (2023) Multimodality imaging assessment of tetralogy of fallot: from diagnosis to Long-Term Follow-Up. Child (Basel) 10(11):1747 Published 2023 Oct 27. 10.3390/children10111747 10.3390/children10111747 PMC10670209 38002838

[12] Voortman M, Fitski W, van Es J et al (2024) Intra- and interobserver reliability in measuring aortic root rotation with cardiac magnetic resonance imaging. Cardiovasc Diagn Ther 14(2):264–271. 10.21037/cdt-23-384 38716314 10.21037/cdt-23-384 PMC11070995

[13] Koo TK, Li MY (2017) A Guideline of Selecting and Reporting Intraclass Correlation Coefficients for Reliability Research [published correction appears in J Chiropr Med.;16(4):346. 10.1016/j.jcm.2017.10.001]. J Chiropr Med. 2016;15(2):155–163. doi:10.1016/j.jcm.2016.02.012 10.1016/j.jcm.2016.02.012 PMC4913118 27330520

[14] Romeih S, Kaoud A, Hashem M et al (2021) A quantitative assessment of aorta root rotation in patients with tetralogy of fallot evaluated by MSCT. Sci Rep 11(1):14336. 10.1038/s41598-021-93814-4. Published 2021 Jul 12 34253813 10.1038/s41598-021-93814-4 PMC8275787

[15] Moradi M, Mirfasihi RS (2020) Is there any association between aortic root rotation angle and aortic dissection? Indian J Thorac Cardiovasc Surg 36(3):181–185. 10.1007/s12055-019-00859-2 33061123 10.1007/s12055-019-00859-2 PMC7525551

[16] Miazza J, Winkel D, Thieringer F, Reuthebuch O, Eckstein F, Gahl B, Berdajs D Aortic root rotation morphological analysis of the aortic root with 3dimensional computed tomography. Eur J Cardiothorac Surg. 2024 Feb 3:ezae040. 10.1093/ejcts/ezae040. Epub ahead of print. PMID: 38310332 10.1093/ejcts/ezae040 PMC10931524 38310332

[17] Lyon SM, Ofner S, Cheng P et al (2023) Serial magnetic resonance imaging for aortic dilation in tetralogy of fallot with pulmonary stenosis. Am J Cardiol 191:92–100. 10.1016/j.amjcard.2022.12.015 36669383 10.1016/j.amjcard.2022.12.015 PMC10337873

[18] Kim W, Kwak JG, Cho S, Kim WH (2023) Ten-year follow-up of dilatation of aortic structures in Fallot-type anomalies. Pediatr Cardiol 44(7):1552–1559. 10.1007/s00246-023-03225-7 37405457 10.1007/s00246-023-03225-7

[19] Tan JL, Davlouros PA, McCarthy KP, Gatzoulis MA, Ho SY (2005) Intrinsic histological abnormalities of aortic root and ascending aorta in tetralogy of fallot: evidence of causative mechanism for aortic dilatation and aortopathy. Circulation 112(7):961–968. 10.1161/CIRCULATIONAHA.105.537928 16087793 10.1161/CIRCULATIONAHA.105.537928

[20] Chowdhury UK, Avneesh S, Ray R et al (2018) A comparative study of histopathological changes in the ascending aorta and the risk factors related to histopathological conditions and aortic dilatation in patients with tetralogy of fallot and a functionally univentricular heart. Heart Lung Circ 27(8):1004–1010. 10.1016/j.hlc.2017.08.011 29111162 10.1016/j.hlc.2017.08.011

[21] Schäfer M, Browne LP, Morgan GJ et al (2018) Reduced proximal aortic compliance and elevated wall shear stress after early repair of tetralogy of fallot. J Thorac Cardiovasc Surg 156(6):2239–2249. 10.1016/j.jtcvs.2018.08.081 30449579 10.1016/j.jtcvs.2018.08.081

[22] Schäfer M, Mawad W (2023) Advanced imaging technologies for assessing tetralogy of fallot: insights into flow dynamics. CJC Pediatr Congenit Heart Dis 2(6Part A):380–392 Published 2023 Oct 5. 10.1016/j.cjcpc.2023.09.011 38161669 10.1016/j.cjcpc.2023.09.011 PMC10755841

[23] Oishi K, Arai H, Oi K et al (2022) The rotational position of the aortic valve: implications for valve-sparing aortic root replacement [published correction appears in Eur J cardiothorac surg. 2022;62(2):ezac276. Doi: 10.1093/ejcts/ezac276]. Eur J Cardiothorac Surg 62(3):ezac179. 10.1093/ejcts/ezac179 35293582 10.1093/ejcts/ezac179

[24] Anderson RH, Spicer DE, Sánchez-Quintana D, Macias Y, Kapadia S, Tretter JT (2023) Relationship between the aortic root and the atrioventricular conduction axis. Heart 109(24):1811–1818 Published 2023 Nov 27. 10.1136/heartjnl-2023-322716 37400231 10.1136/heartjnl-2023-322716

[25] Tretter JT, Spicer DE, Macías Y et al (2023) Vulnerability of the ventricular conduction axis during transcatheter aortic Valvar implantation: A translational pathologic study. Clin Anat 36(5):836–846. 10.1002/ca.24032 36864653 10.1002/ca.24032

[26] Kottner J, Audigé L, Brorson S, Donner A, Gajewski BJ, Hróbjartsson A, Roberts C, Shoukri M, Streiner DL (2011) Guidelines for reporting reliability and agreement studies (GRRAS) were proposed. J Clin Epidemiol 64(1):96–106 Epub 2010 Jun 17. PMID: 21130355 21130355 10.1016/j.jclinepi.2010.03.002

